# Violent Offending Promotes Appetitive Aggression Rather than Posttraumatic Stress—A Replication Study with Burundian Ex-Combatants

**DOI:** 10.3389/fpsyg.2015.01755

**Published:** 2015-12-08

**Authors:** Anke Köbach, Corina Nandi, Anselm Crombach, Manassé Bambonyé, Britta Westner, Thomas Elbert

**Affiliations:** ^1^Clinical and Neuropsychology Group, Department of Psychology, University of KonstanzKonstanz, Germany; ^2^Vivo InternationalKonstanz, Germany; ^3^Department of Clinical Psychology, Université Lumière de BujumburaBujumbura, Burundi

**Keywords:** ex-combatant, soldier, demobilization, DDR, DRC, aggression, PTSD, violence

## Abstract

Research has identified appetitive aggression, i.e., the perception of committed, violent acts as appealing, exciting and fascinating, as a common phenomenon within populations living in precarious and violent circumstances. Investigating demobilized soldiers in the Democratic Republic of Congo (DRC) demonstrated that violent offending is associated with appetitive aggression and not necessarily with symptoms of posttraumatic stress. In the present study, we sought to replicate these results in an independent and larger sample of demobilized soldiers from Burundi. As with the Congolese ex-combatants, random forest regression revealed that the number of lifetime perpetrated violent acts is the most important predictor of appetitive aggression and the number of lifetime experienced traumatic events is the main predictor for posttraumatic stress. Perpetrated violent acts with salient cues of hunting (pursuing the victim, the sight of blood, etc.) were most predictive for perceiving violent cues appealingly after demobilization. Moreover, the association of violent acts and appetitive aggression as well as traumatic events and posttraumatic stress remains strong even years after demobilization. Patterns of traumatic events and perpetrated acts as predictors for posttraumatic stress and appetitive aggression seem to be robust among different samples of ex-combatants who fought in civil wars. Psychotherapeutic interventions that address these complementary facets of combat-related disorders—namely, posttraumatic stress and appetitive aggression—are indispensable for a successful reintegration of those who fought in armed conflicts and to achieve a successful transition to peace.

## 1. Introduction

Combatants who fight in the current civil wars are exposed to numerous forms of extreme violence. They often witness, experience and also perpetrate acts like killing, torture, rape, and other violent attacks. The exposure to combat has consistently been associated with heightened risks for posttraumatic stress disorder (PTSD; Dohrenwend et al., 2006; Hoge et al., 2006; Odenwald et al., 2009; Schaal et al., 2012; Priebe et al., 2013). In the dynamics of scientific research, PTSD is widely considered as disorder of memory where sensory, interoceptive, cognitive, and emotional cues associated with the traumatic event(s) are highly cohesive and insufficiently innervated by contextual information (Ehlers and Clark, 2000; Brewin et al., 2010; Schauer et al., 2011; Elbert et al., 2015). Therefore, anchoring traumatic cues into autobiographic structures using exposure techniques had been proven highly effective during the last decades (Powers et al., 2010; Robjant and Fazel, 2010; Elbert et al., 2015). Recent literature however demonstrated that these interventions were less effective in veteran populations (Steenkamp and Litz, 2013) and that the offender status may hamper treatment gains (Stenmark et al., 2014).

### 1.1. Combat high or traumatic stress

Researchers have been demonstrating that trauma symptoms in veterans are particularly prominent among those who have participated in killing, or have actively been implicated in atrocities (MacNair, 2002; Maguen et al., 2009, 2010; Komarovskaya et al., 2011; Van Winkle and Safer, 2011). Therefore, they categorized perpetration as traumatic (MacNair, 2002). Accordingly, heightened levels of aggression have been found in military personnel exposed to combat (Hecker et al., 2012; Morland et al., 2012) and in some cases, violent outbursts may be explained by the hypervigilance symptoms of posttraumatic stress disorder (Morland et al., 2012). However, perpetrating violent acts might also be perceived appealingly (Nell, 2006; Elbert et al., 2010); a concept termed appetitive aggression (Elbert et al., 2010).

### 1.2. Appetitive aggression

Aggression is commonly defined as overt behavior that has the intention of inflicting physical harm (Anderson and Bushman, 2002). The concept covers two different subtypes: impulsive-reactive and controlled-instrumental (Vitiello and Stoff, 1997; Anderson and Bushman, 2002; Nell, 2006; Elbert et al., 2010). Reactive aggression refers to all aggressive behavior that is provoked by an external trigger. In contrast, instrumental aggression is exerted to achieve a certain goal and—if the behavior was successful—is followed by a positive outcome (Vitiello and Stoff, 1997). Appetitive aggression is a subcategory of the latter; it is exerted to gain a positive feeling and thus, is intrinsically motivated. For the trait of appetitive aggression, cues associated with violent acts are perceived appealingly rather than traumatically (Elbert et al., 2010). The enjoyment of violence in men had been postulated in psychological research before; for instance, Nell (2006) described “the emotional state of the warrior in combat” as “that of predators and hunters, with high arousal, positive affect, and heightened libido (…).” In Grossman (1995) a need for aggression is defined as “combat addiction,” which develops from preceding “combat highs” where the combatant was “float[ing] around, laughing, joking, having a great time, totally oblivious to the dangers around” him. Recently, Moran et al. (2014) demonstrated distinct neural circuitries for appetitive vs. reactive aggression.

Elbert et al. (2010) framed the etiological concept of appetitive aggression as an analog to the theory of *de*-contextualized neural representations in PTSD: as perpetrated violent acts, and thus combat highs accumulate the memory develops a strong but fragmented associative collection of cues (interoceptive, sensory, cognitive, emotional) that are gathered at different points in time. As with traumatic events, context-related neural structures are assumed to be less active in combat highs, and thus implicit/hot and explicit/cold/context information will later be represented by insufficiently connected memory structures. In contrast to the trauma network, the so-called *hunting* network contains however a positive valence (Elbert et al., 2010, 2015).

The exposure of violence—regardless whether witnessed, experienced or perpetrated—shares a considerable number of cues (e.g., blood, suffering, screaming, knife, weapon, or heart beat), which may be associated with both trauma and/or perpetration. Therefore, the *hunting* and the *trauma* networks are linked–they do not however merge due to their opposing valence. According to the context and the current brain state, external stimuli can trigger one or the other network, and thus the corresponding cognitions, emotions, interoception, behavior and/or sensory perception. The line between posttraumatic stress and appetitive aggression blurs with each additional ambivalent cue that is associated with traumatic stress and combat high. Due to the potential domination of shared cues by the hunting network, appetitive aggression may have a preventive effect on posttraumatic stress. This had been demonstrated in various studies (e.g., Weierstall et al., 2011). This antagonistic effect seems to be constrained to patients with low to moderate PTSD symptom severity. After a certain threshold of traumatic events, even people with high appetitive aggression will succumb to PTSD (Weierstall et al., 2013a).

During the last 5 years, the concept has been validated in various countries, including Germany (Weierstall et al., 2012a), South Africa (Weierstall et al., 2013b), DRC (Hecker et al., 2012), Burundi (Crombach and Elbert, 2015), Uganda (Weierstall et al., 2012b), Rwanda (Weierstall et al., 2011), and Colombia (Weierstall et al., 2013a). In sum, the major findings demonstrate that (1) heightened levels of appetitive aggression are common in former combatants/veterans (Weierstall and Elbert, 2011), even years after their demobilization (Weierstall et al., 2012a; Nandi et al., in press), (2) a building block effect for appetitive aggression, i.e., the more types of violent acts are committed, the higher the level of appetitive aggression (Hecker et al., 2012; Crombach et al., 2013; Hermenau et al., 2013a) (see also the building block effect for posttraumatic stress; Schauer et al., 2003), (3) a heightened level of appetitive aggression has a protective impact on posttraumatic stress (Weierstall et al., 2011, 2012a,b; Hecker et al., 2013)—up to a certain threshold of PTSD symptom severity (Weierstall et al., 2013a), and (4), higher ranks in the armed group seem to be associated with a stronger tendency toward violence (Crombach et al., 2013).

### 1.3. Replication

In an attempt to account for reports of combat high and its etiology, one of our earlier studies (Köbach et al., 2014) in the eastern DRC assessed recently demobilized Congolese ex-combatants and found perpetrated violent acts with salient cues of hunting (e.g., attacking a village or settlement, participating in a massacre, etc.) to be the most important predictors for appetitive aggression compared to other specific events or acts. Furthermore, we found evidence that violent offending was significantly associated with appetitive aggression, but only weakly predictive for symptoms of posttraumatic stress (Köbach et al., 2014). Nevertheless, neither events of traumatic stress nor acts of perpetration were totally negligible for the level of appetitive aggression or posttraumatic stress.

Focusing on the overlap between victimization and perpetration, we sought to replicate the results in an independent and larger sample. To examine the association of violent acts and traumatic events with appetitive aggression and posttraumatic stress, we analyzed data from formally demobilized Burundian ex-combatants. A priori hypotheses were set out according to the major findings in DRC (Köbach et al., 2014): (1) Specific types of traumatic events and perpetrated acts (specTE/PA) and the total number of types of traumatic events, witnessed (totTE-wit), experienced (totTE-exp) and perpetrated (totPA), predict the level of appetitive aggression and posttraumatic stress. Perpetrated violence with salient cues of hunting show higher predictive importance than other events. (2) The total number of perpetrated types of violent acts (totPA) is the best predictor for appetitive aggression, while the total number of self-experienced traumatic event types (totTE-exp) is the best predictor for posttraumatic stress.

## 2. Materials and methods

### 2.1. Participants and procedure

We interviewed 392 male Burundian ex-combatants, who were contacted through an official national veteran association. A total of 24 participants had to be excluded due to missing data and one because of invalid answers; the final sample (*N* = 367) had an average age of 36 years (*SD* = 8.5, range: 19–62) and reported 7 years (*SD* = 3.0, range: 0–17) of formal education. On average, they had been recruited by the age of 19 years (*SD* = 4.2, range: 6–39), had spent 12 years (*SD* = 7.3, range: 0–35) in a (para)military group and had been demobilized 5.5 years (*SD* = 2.0, range: 0–14) before we conducted the interviews.

The ethical review boards of the University of Konstanz, Germany and of the University Lumière of Bujumbura, Burundi approved this study. Participation was voluntary and participants had to sign an informed consent prior to the interview (including an explanation of confidentiality requirements in psychological research and therapy). In case of illiteracy, oral informed consents were collected. Fulfilling the highest and most secure data encryption standards, a new electronic data coding and storage procedure using tablet-PCs (iPad) ensured confidentiality. Participants received compensation equivalent to 5 Euro to cover transportation expenses.

Interviews were conducted at the campus of the University Lumière in Bujumbura and took on average 1.5 h. Five clinical psychologists from the University of Konstanz, one clinical psychologist and six advanced students of clinical psychology from the University Lumière interviewed the participants. Interviews were carried out in Kirundi. Non-local interviewers conducted the interviews with the help of five interpreters. Before the application of the interview, all questionnaires had been translated into Kirundi using blind, back and forth translations and were intensively discussed with local experts to guarantee a precise interpretation. All interviewer and interpreters had been trained in the concepts of mental disorders and forms of aggressive behavior prior to the data collection and received continuous supervision to ensure data quality.

### 2.2. Measures

All instruments were applied as a semi-structured interview beginning with the informed consent. In the following first part of the interview, socio-demographic information was collected including age, former (para)military affiliation, age of recruitment, and the years spent in armed groups. Afterwards, exposure to violence, posttraumatic stress, and appetitive aggression were assessed (Nandi et al., in press).

#### 2.2.1. Exposure to violence

**Figure 1:**
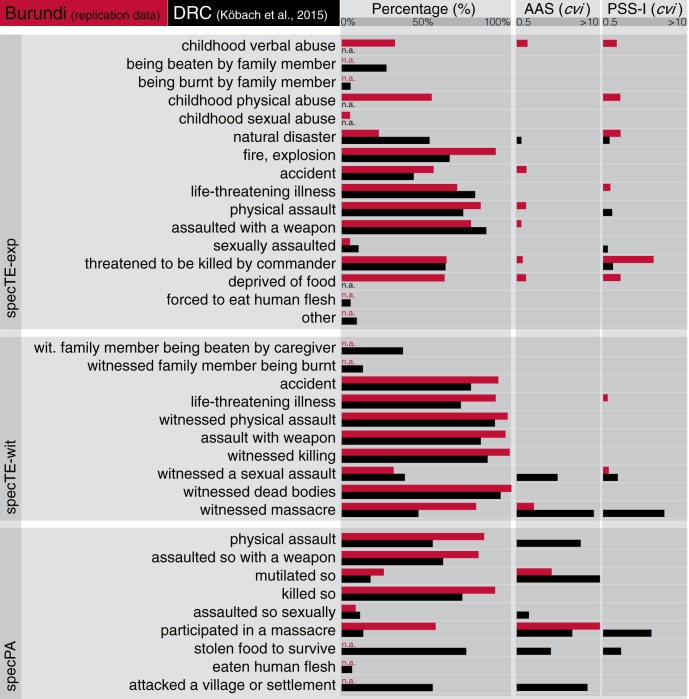
**Lifetime exposure to violence (%) and *cvi*s for the AAS and PSS-I sum scores resulting from ex-combatants in Burundi vs. DRC**. The figure shows similar patterns of prevalence rates as well as the predictor's importances in predicting AAS and PSS-I for Burundian and Congolese ex-combatants. The *cvi*s reveal violent acts with salient cues of hunting (e.g., participating in a massacre) to be most predictive for the AAS score. The prediction of PSS-I by *participation in a massacre* and *stolen food to survive* was not replicated. specTE-exp, specific traumatic events–experienced; specTE-wit, specific traumatic events–witnessed; specPA, specific perpetrated acts; n.a. (red), not assessed in Burundi; n.a. (black), not assessed in DRC.

We assessed exposure to distinct types of violent and potentially traumatic events with a dichotomous (yes/no) checklist of 31 items (nine lifetime self-experienced potentially traumatic events, seven potentially traumatic childhood experiences, nine lifetime witnessed potentially traumatic events, six lifetime perpetrated violent acts; Nandi et al., in press). Aiming for consistency with the original study (Köbach et al., 2014), we excluded five events that had not been assessed in DRC (deprived of food during childhood, social exclusion during childhood, neglect during childhood, loss of caregiver during childhood, witnessing suicide). By summing up the remaining items (26 in total), we calculated the total number of witnessed traumatic event types (totTE-wit; possible range: 0–8) and experienced traumatic event types (totTE-exp; possible range: 0–12), and the number of types of perpetrated violent act (totPA; possible range: 0–6). Furthermore, we used each of the 26 specific traumatic events and perpetrated acts (specTE/PA) as predictors. Figure 1 provides an overview of the events included in the analysis of data assessed in Burundi and DRC and their incidence rates.

#### 2.2.2. Posttraumatic stress

Symptoms of PTSD were investigated using the PTSD Symptom Scale—Interview (PSS-I, Foa et al., 1993; Foa and Tolin, 2000). The PSS-I is a semi-structured interview, which consists of 17 items corresponding to the diagnostic criteria of the DSM-IV and referring to the last 4 weeks. Each item is rated on a four-point scale ranging from 0 (*not at all/only once*) to 3 (*five or more times per week/almost always*). The instrument has proven validity in comparable East-African samples (Ertl et al., 2010). In the present study Cronbach's α was 0.93.

#### 2.2.3. Appetitive aggression

To assess appetitive aggression we used the Appetitive Aggression Scale (AAS; Weierstall and Elbert, 2011), a semi-structured interview that has also been used and validated in comparable populations. The AAS consists of 15 items rated on a five-point scale ranging from 0 (*I totally disagree*) to 4 (*I totally agree*). Appetitive aggression is conceptualized as trait, thus referring to a positive perception of violence (e.g., “Is it exciting for you if you make an opponent really suffer?,” “Once fighting has started do you get carried away by the violence?,” or “Once you got used to being cruel, did you want to be crueller and crueller?”). In the present study Cronbach's α was 0.89.

### 2.3. Analysis

As in the original study (Köbach et al., 2014), we used random forest regression with conditional inference trees (RF-CI), a non-parametric machine learning technique. Unlike the classical random forest, the RF-CI does not display a bias toward predictors with many categories in the variable selection process (Strobl et al., 2008). Following the principles of ensemble methods, a certain number of trees are aggregated to compose the random forest. Each tree is built using binary splits of the previously subsampled data (subsampling rate = 63.2%; Strobl, 2008; Strobl et al., 2009a). The splitting variable is chosen according to the strength of the association between the covariates and the outcome (Hothorn et al., 2006b; Strobl et al., 2009b) from a set of randomly preselected predictors (Grömping, 2009). Next, the importance of each predictor variable is ranked based on the ensemble of trees (conditional variable importance, *cvi*; Strobl et al., 2008). The goodness of fit can be assessed using the out-of-bag data (OOB). The results are used to calculate a pseudo-*R*^2^ from the mean squared error (MSE) and the total sum of squares (SST; *OOB-R*^2^ = 1–MSE/SST; Grömping, 2009).

RF-CI provides an ordinal ranking of variables according to their importance for the outcome prediction (conditional variable importance, *cvi*), thus in this study for traumatic events and perpetrated acts in order to predict the level of appetitive aggression and posttraumatic stress. Further, the OBB-*R*^2^ implies the fit of the model (Strobl et al., 2009b). Consequently, four models are presented in this study: (1) the AAS sum score predicted by specTE/PA (RF-CI:1, spec-model_*AAS*_), (2) the AAS sum score predicted by totTE-exp, totTE-wit, and totPA (RF-CI:2, tot-model_*AAS*_), (3) The PSS-I sum score predicted by specTE/PA (RF-CI:3, spec-model_*PSS*−*I*_), (4) PSS-I sum score predicted by totTE-exp, totTE-wit, and totPA (RF-CI:4, tot-model_*PSS*−*I*_). The analysis was conducted using R (version 2.15.0); the implementation we used was cforest (Hothorn et al., 2006a) from the R package party (Strobl et al., 2009a) with unbiased variable selection (Hothorn et al., 2006a,b, following Westner, 2013).

Furthermore, Brandt et al. (2014) recently published a “replication recipe” including crucial criteria for such endeavors. This guideline provides an efficient and standardized way to present the importance of a replication, the methods, and the results. The “36-question guide of the Replication Recipe” was applied in the present study (see Table 1 at the end of the article).

**Table 1:** **Thirty-six-question guide to the Replication Recipe (Brandt et al., 2014)**.

**THE NATURE OF THE EFFECT**		
1	Verbal description of the effect I am trying to replicate:	(a) Cumulative lifetime perpetrated acts of violence are associated with higher levels of appetitive aggression.(b) Particularly, events that incorporate salient cues of violence have higher predictive importance for appetitive aggression
2	It is important to replicate this effect because …	…the appealing aspect of violence is often neglected although it has an immense impact on (post-war) societies and mostly co-occurs with poor mental health
3	The effect size of the effect I am trying to replicate is:	n/a; As in the original study we used RF-CI. Outcome measures can be drawn from Figure 1 in the article
4	The confidence interval of the original effect is:	n/a
5	The sample size of the original effect is:	*N* = 95
6	Where was the original study conducted? (e.g., lab, in the field, online)	In the field
7	What country/region was the original study conducted in?	Democratic Republic of Congo, North Kivu, Goma
8	What kind of sample did the original study use? (e.g., student, Mturk, representative)	Demobilizing, male, adult, Congolese ex-combatants
9	Was the original study conducted with paper-and pencil surveys, on a computer, or something else?	The original study was conducted with paper-and pencil. The replication study was conducted with iPads
**DESIGNING THE STUDY**		
10	Are the original materials for the study available from the author?	On demand
11	I know that assumptions (e.g., about the meaning of the stimuli) in the original study will also hold in my replication because:	Yes.
12	Location of the experimenter during data collection:	On site
13	Experimenter knowledge of participant experimental condition:	Interviewers did not know the hypothesis
14	Experimenter knowledge of overall hypotheses:	Interviewers did not know the hypothesis
15	My target sample size is:	n/a
16	The rationale for my sample size is:	Interview capacity, time, availability of ex-combatants
**DOCUMENTING DIFFERENCES BETWEEN THE ORIGINAL AND REPLICATION STUDY**		
For each part of the study indicate whether the replication study is *Exact, Close*, or *Conceptually Different* compared to the original study. Then, justify the rating		
17	The similarities/differences in the instructions are:	[Exact]
18	The similarities/differences in the measures are:	[Close]; few events/acts were not asked in the replication study and vice versa. Outcome (AAS, PSS-I sum scores) was measured by exactly the same instruments
19	The similarities/differences in the stimuli are:	n/a
20	The similarities/differences in the procedure are:	[Close]; iPads were used documenting the answers in the replication study. The interview setting was the same, but in different countries
21	The similarities/differences in the location (e.g., lab vs. online; alone vs. in groups) are:	[Exact]
22	The similarities/differences in remuneration are:	[Exact]; transport money was paid in the replication study; in the original study ex-combatants were on site and thus no transport necessary
23	The similarities/differences between participant populations are:	[Close]; similarities: ex-combatants in both studies fought in one or more African civil wars with similar kinds of events and living conditions; differences: ex-combatants in DRC have been in their demobilization process, while those in Burundi had been demobilized on average 6 years ago
24	What differences between the original study and your study might be expected to influence the size and/or direction of the effect?	In the replication study participants had been demobilized for a longer period of time
25	I have taken the following steps to test whether the differences listed in #24 will influence the outcome of my replication attempt:	n/a
**ANALYSIS AND REPLICATION EVALUATION**		
26	My exclusion criteria are (e.g., handling outliers, removing participants from analysis):	Participants with missing(s) in the event list, PSS-I or AAS as well as participants who gave invalid answers were excluded from the analysis
27	My analysis plan is (just if it differences from the original):	n/a
28	A successful replication is defined as:	(a) Similar pattern of variable importance (*cvi*s) and (b) acceptable model fits
**REGISTERING THE REPLICATION ATTEMPT**		
29	The finalized materials, procedures, analysis plan, etc. of the replication are registered here:	In the main article
**REPORTING THE REPLICATION**		
30	The effect size of the replication is:	n/a
31	The confidence interval of the replication effect size is:	n/a
32	The replication effect size [is/is not] (circle one) significantly different from the original effect size?	n/a
33	I judge the replication to be a(n) [success/informative failure to replicate/practical failure to replicate/inconclusive] (circle one) because:	Success; The patterns of variance importances (*cvi*s) are similar and the model fits acceptable
34	Interested experts can obtain my data and syntax here:	On demand
35	All of the analyses were reported in the report or are available here:	In the article. Further information about the Burundian sample are published in Nandi et al. (in press)
36	The limitations of my replication study are:	Few events were not asked in the replication study and vice versa

## 3. Results

### 3.1. Exposure to violence

The participants were exposed to a high range of experienced (*M* = 6.1, *SD* = 2.0, range: 0–12) and witnessed (*M* = 6.8, *SD* = 1.0, range: 2–8) traumatic event types, and perpetrated violent acts (*M* = 3.4, *SD* = 1.4, range: 0–6). Only 16 (4.4 %) participants reported having committed none of the perpetrated violent acts. Everyone had witnessed at least two traumatic event types, and only one participant reported not having experienced any of the traumatic events. The high variance of combat exposure is also reflected in the levels of appetitive aggression (*M* = 28.5, *SD* = 14.6, range: 0–58) and posttraumatic stress (*M* = 13.7, *SD* = 11.2, range: 0–42).

### 3.2. Specific traumatic events and perpetrated acts (RF-CI:1 and RF-CI:3)

Figure 1 illustrates the *cvi*s for the AAS and PSS-I sum scores in comparison with the original study. The OBB-*R*^2^ explained 36% of variance for the AAS and 30% for the PSS-I sum score (in the Congolese sample of the original study, 33% of the variance was explained for the AAS and 27% for the PSS-I sum score).

### 3.3. Total scores of events and acts (RF-CI:2 and RF-CI:4)

The pattern of the *cvi*-values of the total scores was similar in Burundian ex-combatants, compared to Congolese ex-combatants. TotPA had the highest predictive value for the AAS sum score (*cvi*_*Burundi*_ = 28, *cvi*_*DRC*_ = 88), compared to totTE-exp (*cvi*_*Burundi*_ = 13, *cvi*_*DRC*_ = 4) and totTE-wit (*cvi*_*Burundi*_ = 5, *cvi*_*DRC*_ = 4). TotTE-exp (*cvi*_*Burundi*_ = 24, *cvi*_*DRC*_ = 10) had the highest impact on participants' PSS-I sum scores; totTE-wit had a minor impact (*cvi*_*Burundi*_ ≤ 1, *cvi*_*DRC*_ = 8). Essentially, the importance of the totPA in predicting the PSS-I sum score was low in both studies: *cvi*_*Burundi*_ = 1 and *cvi*_*DRC*_ = 2. The OBB-*R*^2^ explained 37% of the variance for the AAS sum score (RF-CI:2) and 24% for the PSS-I sum score (RF-CI:4); (in the Congolese sample, 44% of the variance was explained for the AAS and 34% for the PSS-I sum score).

## 4. Discussion

In this article, we successfully replicated the most important findings presented in a previous study with Congolese ex-combatants. With a larger sample of Burundian ex-combatants, we showed that specific types of traumatic events and violent acts, as well as the total number of these incidents, predicted appetitive aggression and posttraumatic stress. Moreover, the total number of perpetrated violent acts (totPA) was the most important predictor for the level of appetitive aggression, while the total number of experienced traumatic event types (totTE-exp) was the most important predictor for posttraumatic stress. Specific event types and violent acts with salient cues of violence had the strongest impact on the level of appetitive aggression in both samples, which thereby seems to be a robust finding. The accumulated number of violent acts and traumatic event types (tot-model_*AAS*∕*PSS*−*I*_) was not superior in predicting the level of appetitive aggression or posttraumatic stress, in comparison to models that used specific events (spec-model_*AAS*∕*PSS*−*I*_).

The findings in this article strengthen the evidence that perpetrated violent acts can be perceived as appealing (Elbert et al., 2010), thus not as traumatic (MacNair, 2002) and most importantly, are rather associated with appetitive aggression (*cvi*_*Burundi*_ = 28, *cvi*_*DRC*_ = 88) than with posttraumatic stress (*cvi*_*Burundi*_ = 1, *cvi*_*DRC*_ = 2). In addition, none of the specific perpetrated acts revealed important predictive value for predicting posttraumatic stress in this replication study. Nevertheless, traumatic events seem relevant for predicting appetitive aggression. This became particularly obvious in the current study. For psychotherapeutic interventions with individuals previously involved in violent conflicts it is imperative to abandon the dichotomous victim-perpetrator archetype. The symptoms of posttraumatic stress and heightened levels of aggression can be addressed and treated more effectively when all forms of exposure to violence—experienced, witnessed, and perpetrated—as well as the associated feelings are taken into consideration without judgment. Such an attempt has been made in Narrative Exposure Therapy for Forensic Offender Rehabilitation (FORNET; Elbert et al., 2012; Hermenau et al., 2013b; Crombach and Elbert, 2015; Köbach et al., in press). In 5–7 sessions, the therapist and the client intensively reflected on the most poignant incidents of violence (exposure in sensu) and framed crucial, transitory changes from combatant to civilian. FORNET successfully reduced posttraumatic stress (Hermenau et al., 2013b; Köbach et al., in press) and the number of violent offenses (Crombach and Elbert, 2015). Further research is required.

Furthermore, this study replicated the results that “participation in a massacre,” “having mutilated someone,” and “having witnessed a massacre”—events/acts with very salient and predatory cues of violence (e.g., blood, screaming, suffering, etc.)—are important predictors for the level of appetitive aggression. In the present study, this was the case even years after demobilization! These events may deserve particular focus in psychotherapy.

The discrepancy in the time difference since demobilization in the current sample (6 years) in comparison to the Congolese sample in which the participants were interviewed during the demobilization process, suggests that the associations between perpetrated acts and appetitive aggression as well as traumatic events and posttraumatic stress remain stable over time; in fact, the patterns of variable importances are astonishingly similar, both for single specific event types/acts as well as the total exposure to different forms of violence (see Figure 1). The model fits were generally lower in the replication study.

This replication article has limitations. First, the earlier result indicating that the total number of lifetime traumatic event types and violent acts would predict PTSD better than specific event types was not replicated for both the level of appetitive aggression and posttraumatic stress. Further, the models were not exactly the same as were used in the original study, owed to the slightly varying event lists. Finally, it is noteworthy, that causal interpretations cannot be drawn given the cross-sectional study design together with a rather descriptive statistical operationalization.

## 5. Conclusion

In conclusion, the results indicate that the perpetration of violent acts during war continuously shapes a perpetrator's perception of and affiliation for violence. To create stable communities, secure family environments and healthy minds, evidence-based mental health care needs to abandon simplistic victim-perpetrator dichotomies and develop interventions that take the whole range of combat-related complications into account.

## Author contributions

CN and AC performed the interviewer training and supervised the data collection. MB provided support on site. AK performed the data analysis and interpretation with support by BW and under the supervision of TE. AK drafted the paper, and CN, AC, and TE provided critical revisions. All of the authors approved the final version of the paper for submission.

## Funding

VolkswagenStiftung und Deutsche Forschungsgesellschaft.

### Conflict of interest statement

The authors declare that the research was conducted in the absence of any commercial or financial relationships that could be construed as a potential conflict of interest.
